# Discovery of Novel Mono-Carbonyl Curcumin Derivatives as Potential Anti-Hepatoma Agents

**DOI:** 10.3390/molecules28196796

**Published:** 2023-09-25

**Authors:** Weiya Cao, Pan Yu, Shilong Yang, Zheyu Li, Qixuan Zhang, Zengge Liu, Hongzhuo Li

**Affiliations:** 1 College of Public Health, Anhui University of Science and Technology, Hefei 230000, China; sml131415@163.com; 2 College of Medicine, Anhui University of Science and Technology, Huainan 232001, China; 17758194831@163.com (Z.L.); 13753738161@163.com (Q.Z.); 15771326234@163.com (Z.L.); 17767778237@163.com (H.L.); 3 College of Chemical Engineering, Nanjing Forestry University, Nanjing 210037, China; yshl6072@njfu.edu.cn

**Keywords:** curcumin derivatives, anti-hepatoma activity, AKT inhibition, molecular docking, xenograft model

## Abstract

Curcumin possesses a wide spectrum of liver cancer inhibition effects, yet it has chemical instability and poor metabolic properties as a drug candidate. To alleviate these problems, a series of new mono-carbonyl curcumin derivatives **G1–G7** were designed, synthesized, and evaluated by in vitro and in vivo studies. Compound **G2** was found to be the most potent derivative (IC_50_ = 15.39 μM) compared to curcumin (IC_50_ = 40.56 μM) by anti-proliferation assay. Subsequently, molecular docking, wound healing, transwell, JC-1 staining, and Western blotting experiments were performed, and it was found that compound **G2** could suppress cell migration and induce cell apoptosis by inhibiting the phosphorylation of AKT and affecting the expression of apoptosis-related proteins. Moreover, the HepG2 cell xenograft model and H&E staining results confirmed that compound **G2** was more effective than curcumin in inhibiting tumor growth. Hence, **G2** is a promising leading compound with the potential to be developed as a chemotherapy agent for hepatocellular carcinoma.

## 1. Introduction

Curcumin (Curc), a well-known active polyphenolic component from the rhizome of the plant *Curcuma longa*, demonstrates a variety of pharmacological properties, particularly anticancer activity, which has attracted widespread interest among medicinal chemists in recent years [1,2,3]. Curcumin is being developed as a potential anti-hepatoma drug, although there have been absorption issues. One of the primary causes of chemical instability and poor metabolic characteristics, which lead to low bioavailability and limited therapeutic value, is the instability of the β-diketone group in the curcumin structure [4,5]. The curcumin β-diketone structure has undergone some changes, including the retention of the aryl rings’ 3-, 5-, and 7-carbon spacers [6,7,8,9,10,11,12,13,14,15,16,17].

**Figure 1:**
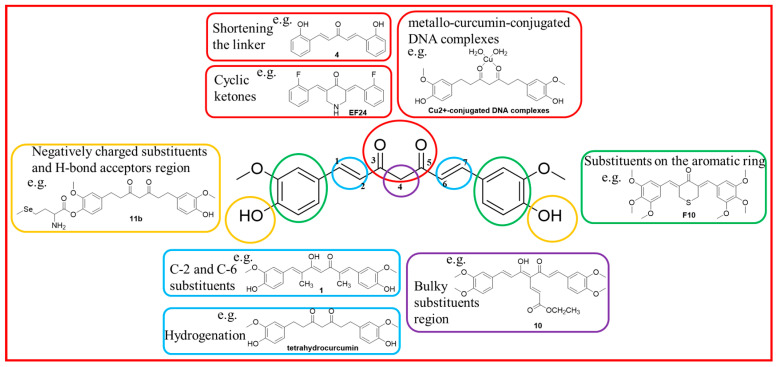
The SAR analysis and the structures of the most important compounds.

The structure–activity relationship (SAR) study of curcumin derivatives could be concluded as shown in Figure 1: (1) shortening the linker from seven carbon atoms (C7) to five carbon atoms (C5) or three carbon atoms (C3) can improve the stability and anticancer activity, as demonstrated by compound **4** [6,18,19]; (2) introducing a five- or six-carbon ring or a heterocyclic ring (N, O, and S atoms) into the β-diketone moiety can increase molecular rigidity and improve anticancer activity, as is the case for compound **EF24**, which is now in clinical trials [20]; (3) metallo-curcumin-conjugated DNA complexes by using Cu^2+^/Ni^2+^/Zn^2+^ metal ions can improve curcumin solubility and enhance DNA-binding ability, an example being Cu^2+^-conjugated DNA complexes [21]; (4) introducing a methyl group at the C-2 and C-6 positions exhibits a steric hindrance effect toward metabolizing enzymes, such as alcohol dehydrogenase, and demonstrates significantly higher activity than curcumin, as shown by compound **1** [22]; (5) the hydrogenation function of the β-diketone moiety is also important, like in dihydrocurcumin and tetrahydrocurcumin derivatives [23,24]; (6) the presence of a coplanar hydrogen donor group at the C-4 position can improve anticancer activity, exemplified by compound **10** as a potential antiandrogenic agent for the treatment of prostate cancer [25]; (7) masking the 4-OH groups of the benzyl rings enhances curcumin’s water solubility and kinetic stability, as demonstrated by compound **11b** [26,27]; (8) introducing suitable substituents such as a methoxy group on the aromatic ring can also improve the antitumor activity, shown by, for example, the inhibitory activity of compound **F10** against human prostate cancer cell line PC-3, which is 73 times that of curcumin [28].

According to the SAR study, converting the -diketone structure into a single ketone can greatly increase stability and pharmacological activity [7,9,13,14,15]. Additionally, curcumin’s hydroxy on benzene can be changed to improve anticancer biological activity using compounds like mono-carbonyl curcumin cinnamyl ester B5 and dimethyl curcumin (ASC-J9) [29,30,31]. Therefore, the major emphasis of curcumin structural alteration is on the benzene hydroxyl group and the β-diketone structure, which might significantly improve stability, bioavailability, and anticancer activity [32,33,34]. However, the structural diversity is relatively constrained and the underlying mechanism involved in the effect of curcumin is not well elucidated, especially in antitumor activity. In this study, we introduce natural products such as benzoic acid analogs or niacin on the hydroxy of mono-carbonyl curcumin to increase the anti-human hepatocellular carcinoma (HCC) activity.

## 2. Materials and Methods

### 2.1. Chemistry

#### 2.1.1. General

All reagents were analytical reagent grade and purchased from Shanghai Titan Technology Co., LTD. (Shanghai, China). ^1^H NMR and ^13^C NMR spectra were recorded with Bruker AM 600 MHz and 400 MHz spectrometers. Melting points (M.P.) were determined on an SRS OptiMelt-100 instrument and ESI-MS was recorded on an Agilent 6520B Q-TOF system. The purity of the compounds was analyzed by HPLC Agilent 1260 (Agilent, Santa Clara, CA, USA). The injection volume was 50 μL, with flow rate set at 1 mL/min. Reactions were monitored by thin-layer chromatography (TLC) on a glass plate coated with silica gel with the fluorescent indicator (GF254).

#### 2.1.2. Preparation of Curcumin Derivatives **G1**–**G7**

**Figure 2:**
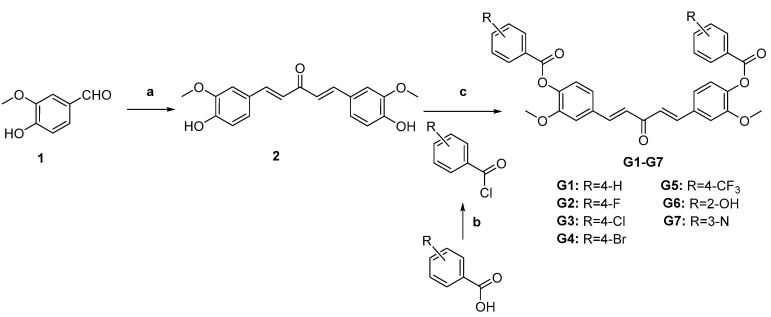
Synthesis of the curcumin derivatives G1–G7. Reagents and conditions: (**a**) acetone, aqueous NaOH, ethanol, 48 h; (**b**) SOCl_2_, 80 °C, reflux, 2 h; (**c**) CHCl_3_, triethylamine, 0 °C to room temperature, 12 h.

The synthesis of pale yellow compound **2** (Figure 2) from 4-hydroxy-3-methoxybenzaldehyde (**1**) was carried out by the same procedure as described in the previous article [31]. The starting materials (benzoic acid analogs and niacin) for the synthesis of esters should be activated in the first procedure: the benzoic acid analogs and niacin (1 mmol) and SOCl_2_ (5 mL) were mixed and stirred at a reflux temperature of 80 °C for 2 h. The reaction mixture was cooled and evaporated to give reactive acyl chloride obtained as a solid or oil, which would be dissolved in CHCl_3_ in the next step. A solution of acyl chloride in CHCl_3_ was added dropwise to intermediate compound **2** (0.3 mmol) in CHCl_3_ containing triethylamine at 0 °C with constant stirring for 12 h. The reaction mixture was then neutralized by adding 1 N HCl and extracted with CH_2_Cl_2_. The combined organic layer was dried over anhydrous sodium sulfate and was purified by flash chromatography with methanol/CH_2_Cl_2_ to give these white compounds **G1**–**G7** (Figure 2). The yields were between 55% and 65%.

#### 2.1.3. Molecular Docking Studies

Molecular docking was performed using the Glide module in Schrodinger 2017 to explore the interaction mode between the tested compounds and AKT protein [35]. The crystal structure of AKT (PDB ID: 3O96) was downloaded from the Protein Data Bank [36]. The initial structures were prepared with the Protein Preparation Wizard workflow and the receptor grid was generated according to the endogenous ligand. The small molecules were optimized with the LigPrep workflow and minimized using the OPLS-2005 force field with the conformational search method. All other parameters were set as default and the binding affinity was evaluated by the binding free energy (Kcal/mol) with standard precision (SP) and extra precision (XP) docking mode. Finally, plausible docking models were selected from the abundant clusters between the ligand and receptor that had lower binding energies, and intermolecular interactions were illustrated using PyMOL 2.5.5 software.

### 2.2. Biological Assays

#### 2.2.1. Cell Culture

Human hepatocellular carcinoma cell line HepG2 was obtained from Nanjing Forestry University. MTT (3-(4,5-Dimethylthiazol-2-yl)-2,5-diphenyltetrazolium bromide) was purchased from Sigma, and 6-well, 24-well, and 96-well plates were purchased from Beyotime Biotechnology. Cells were grown in DMEM medium with 10% fetal bovine serum at 37 °C and 5% CO_2_. All compounds were dissolved in DMSO of pure analytical grade, diluted using DMEM medium, and the final concentration had no effects on cell viability.

#### 2.2.2. MTT Assay

HepG2 cells (5000 cells/well) in the logarithmic phase were treated with 5, 10, and 15 μM curcumin derivatives **G1**–**G7** in 96-well culture plates for 48 h. Then, 20 μL, 4 mg/mL MTT of PBS solution was added to each well. After incubation for 4 h, the medium was carefully removed and formazan crystals were dissolved in 200 μL of DMSO in the dark, and then absorbance values at 490 nm wavelength were read on a Super Microplate Reader (MQX2OO) (BioTek, the United States). Compound **G2** was then selected to treat HepG2 cells with different concentrations (0.5, 1, 2, 4, 8, 16, 32, 64 μM) for 48 h, and IC_50_ values were calculated using GraphPad Prism 8 software. Each assay was performed with at least three independent experiments [37].

#### 2.2.3. Clone Formation Assay

HepG2 cell line (500 cells per well) was incubated in a 6-well plate at 37 °C and 5% CO_2_ for 12 h. Compound **G2** was added containing the indicated concentrations (0, 1, 2, 4 μM) in DMEM medium and then incubated for 2 weeks. Subsequently, the cells were fixed with 4% paraformaldehyde and stained with 0.1% crystal violet solution for about 20 min. The cells were washed with PBS, dried, and photographed, and the clone formation rate was calculated [38].

#### 2.2.4. Wound Healing Assay

HepG2 cells (1.5 × 10^5^ cells/well) were put into 24-well plates and cultured in DMEM to a nearly confluent cell monolayer. Then, the scratch was performed using a 10 μL plastic pipette tip. The debris or detached cells were washed twice with serum-free DMEM medium. The cells were subjected to target compound **G2** at different concentrations (0, 2.5, 5, 10 μM) in fresh DMEM medium and subsequently cultured for 24 h or 48 h. The migration rate was measured and photographed under a DMIL LED 3000 inverted microscope (Leica, Wetzlar, Germany) [39].

#### 2.2.5. Transwell Migration Assay

HepG2 cells (5 × 10^4^ cells/well) were incubated in 200 μL serum-free DMEM in the upper transwell chamber and 700 μL DMEM at the bottom. The cells were treated with **G2** (0, 5 μM) for 48 h. The filter was fixed in 4% paraformaldehyde for 30 min and stained with 0.1% crystal violet for 15 min. The migrated cells on the lower surface were counted three times under an inverted microscope [40].

#### 2.2.6. Mitochondrial Membrane Potential Assay

HepG2 cells were treated with compound **G2** (0, 2.5, 5, 10 μM) in a 6-well plate for 48 h. The cells were washed with PBS and 1 mL JC-1 staining (Beyotime, C2006) was added for 20 min at 37 °C. After being rinsed twice with PBS, the samples were examined by a Nikon fluorescence microscope (TE2000-U, Nikon Inc., Tokyo, Japan) at 40× *g* magnification under 488 nm and 550 nm [31].

#### 2.2.7. Western Blot Analysis

HepG2 cells in 6-well plates were rinsed twice with cold PBS and lysed on ice for 30 min. After centrifugation, the protein concentrations were measured using the BCATM protein quantification kit (Beyotime Biotechnology). Antibodies against AKT (C67E7), Bcl-2 (D55G8), phospho-Bcl-2 (5H2), caspase 3 (D3R6Y), cleaved caspase 3 (5AE1), and β-actin (13E5) were purchased from CST or ABCAM. Briefly, the quantified protein samples were subjected to an SDS-PAGE and then transferred to a PVDF membrane. After incubating with primary antibodies at 4 °C overnight and secondary antibodies at room temperature for 2 h, the membrane was illuminated with ECL solution and the density was analyzed using Image J [41].

#### 2.2.8. Xenograft Model and In Vivo Study

The BALB/c nude mice (female, 6–8 weeks, 18–20 g) were purchased from Nanjing Junke Biological Co., LTD. (Nanjing, China). Animal welfare and experimental procedures were followed according to the Guide for Care and Use of Laboratory Animals (National Institutes of Health, Bethesda, MD, USA) and the system guidance of the Anhui University of Science and Technology Animal Management Committee. Briefly, HepG2 cells were washed with PBS and the suspension (2 × 10^6^ cells in 0.1 mL) was injected into the right flank of nude mice. The treatment was initiated when the tumor volume reached approximately 100 mm^3^ and mice were randomly divided into 3 groups: the control group (5 rats), the curcumin group (5 rats), and the G2 group (5 rats). The compounds at the desired concentration in olive oil were intravenously injected at a frequency of one dose per day. Body weights and tumor volumes were measured every 3 days and tumor volumes were calculated using the following formula: 0.5 × L × (W)^2^, where L and W are the length and width of the tumor mass, respectively [42].

#### 2.2.9. H&E Staining Analysis

Liver tissues from different model groups were isolated and fixed in 4% paraformaldehyde. After being dehydrated using ethanol and xylene, the liver tissue was embedded in paraffin using a tissue embedding machine. Subsequently, the paraffin-fixed tissue samples were sliced to a thickness of 4–5 μm, and hematoxylin and eosin (H&E) staining was performed [43].

## 3. Results and Discussion

### 3.1. Chemistry

The synthesis of target compounds **G1**–**G7** followed the general pathway outlined in Figure 2. All of the synthetic compounds were reported for the first time and given satisfactory analytical and spectroscopic data. ^1^HNMR, ^13^CNMR, and ESI-MS analysis spectra were in full accordance with the assigned structures. The HPLC chromatogram of the compounds was recorded at λ_426_ nm and the purity levels were more than 95%.

(1E,4E)-1,5-bis(4-hydroxy-3-methoxyphenyl)penta-1,4-dien-3-one (**2**).

Calcd. ESI-MS *m*/*z*: 325.3475 (M-H)^−^; measured: 325.3480. M.P.:128–130 °C. ^1^H NMR (400 MHz, DMSO-*d*_6_) δ 9.62 (s, 1H), 7.52 (d, *J* = 16.3 Hz, 1H), 7.30 (s, 1H), 7.13 (d, *J* = 8.2 Hz, 1H), 6.81 (d, *J* = 8.1 Hz, 1H), 6.67 (d, *J* = 16.2 Hz, 1H), 3.82 (s, 3H). ^13^C NMR (101 MHz, DMSO-*d*_6_) δ 197.8, 149.4, 148.0, 143.9, 125.9, 124.3, 123.2, 115.6, 111.3, 55.7 (Figures S1 and S2).

((1E,4E)-3-oxopenta-1,4-diene-1,5-diyl)bis(2-methoxy-4,1-phenylene) dibenzoate (**G1**).

Calcd. ESI-MS *m*/*z*: 533.5636 (M-H)^−^; measured: 533.5640. M.P.:119–121 °C. ^1^H NMR (600 MHz, Chloroform-*d*) δ 8.21 (d, *J* = 7.1 Hz, 2H), 7.65 (t, *J* = 8.0 Hz, 1H), 7.55–7.48 (m, 3H), 7.18 (d, *J* = 10.5 Hz, 3H), 6.69 (d, *J* = 16.3 Hz, 1H), 3.86 (s, 3H). ^13^C NMR (151 MHz, Chloroform-*d*) δ 198.4, 164.64, 151.9, 142.9, 142.1, 133.8, 133.6, 130.5, 129.2, 128.7, 127.5, 123.6, 121.7, 111.5, 56.1 (Figures S3 and S4).

((1E,4E)-3-oxopenta-1,4-diene-1,5-diyl)bis(2-methoxy-4,1-phenylene) bis(4-fluorobenzoate) (**G2**).

Calcd. ESI-MS *m*/*z*: 569.5443 (M-H)^−^; measured: 569.5448. M.P.:120–122 °C. ^1^H NMR (400 MHz, Chloroform-*d*) δ 8.30–8.15 (m, 2H), 7.50 (d, *J* = 16.2 Hz, 1H), 7.18 (d, *J* = 6.6 Hz, 5H), 6.69 (d, *J* = 16.3 Hz, 1H), 3.86 (s, 3H). ^13^C NMR (101 MHz, Chloroform-*d*) δ 198.4, 163.7, 151.8, 142.8, 141.9, 133.7, 133.18, 133.1, 127.5, 125.5, 123.6, 121.7, 116.1, 115.9, 111.5, 56.1 (Figures S5 and S6).

((1E,4E)-3-oxopenta-1,4-diene-1,5-diyl)bis(2-methoxy-4,1-phenylene) bis(4-chlorobenzoate) (**G3**).

Calcd. ESI-MS *m*/*z*: 602.4474 (M-H)^−^; measured: 602.4480. M.P.:110–112 °C. ^1^H NMR (400 MHz, Chloroform-*d*) δ 8.14 (d, *J* = 7.5 Hz, 2H), 7.54–7.46 (m, 3H), 7.18 (d, *J* = 6.4 Hz, 3H), 6.69 (d, *J* = 16.3 Hz, 1H), 3.86 (s, 3H). ^13^C NMR (101 MHz, Chloroform-*d*) δ 198.4, 163.8, 151.8, 142.8, 141.8, 140.4, 133.7, 131.9, 129.1, 127.7, 127.6, 123.6, 121.7, 111.5, 56.1 (Figures S7 and S8).

((1E,4E)-3-oxopenta-1,4-diene-1,5-diyl)bis(2-methoxy-4,1-phenylene) bis(4-bromobenzoate) (**G4**).

Calcd. ESI-MS *m*/*z*: 691.3556 (M-H)^−^; measured: 691.3560. M.P.:123–125 °C. ^1^H NMR (400 MHz, Chloroform-*d*) δ 8.06 (d, *J* = 8.2 Hz, 2H), 7.66 (d, *J* = 8.2 Hz, 2H), 7.50 (d, *J* = 16.2 Hz, 1H), 7.17 (d, *J* = 5.8 Hz, 3H), 6.69 (d, *J* = 16.2 Hz, 1H), 3.85 (s, 3H). ^13^C NMR (101 MHz, Chloroform-*d*) δ 198.3, 163.9, 151.7, 142.8, 141.8, 133.7, 132.1, 132.0, 129.1, 128.1, 127.6, 123.5, 121.7, 111.5, 56.1 (Figures S9 and S10).

((1E,4E)-3-oxopenta-1,4-diene-1,5-diyl)bis(2-methoxy-4,1-phenylene) bis(4-(trifluoromethyl)benzoate) (**G5**).

Calcd. ESI-MS *m*/*z*: 669.5600 (M-H)^−^; measured: 669.5604. M.P.:206–208 °C. ^1^H NMR (400 MHz, Chloroform-*d*) δ 8.33 (d, *J* = 8.0 Hz, 2H), 7.79 (d, *J* = 8.0 Hz, 2H), 7.51 (d, *J* = 16.2 Hz, 1H), 7.19 (d, *J* = 7.6 Hz, 3H), 6.74–6.66 (m, 1H), 3.86 (s, 3H). ^13^C NMR (101 MHz, Chloroform-*d*) δ 198.3, 163.5, 151.7, 142.7, 141.6, 135.4, 135.1, 133.9, 132.5, 130.9, 127.7, 125.8, 125.1, 123.5, 122.3, 121.7, 111.5, 56.1 (Figures S11 and S12).

((1E,4E)-3-oxopenta-1,4-diene-1,5-diyl)bis(2-methoxy-4,1-phenylene) bis(2-hydroxybenzoate) (**G6**).

Calcd. ESI-MS *m*/*z*: 565.5615 (M-H)^−^; measured: 565.5620. M.P.:135–137 °C. ^1^H NMR (600 MHz, Chloroform-*d*) δ 10.37 (s, 1H), 8.09 (d, *J* = 8.0 Hz, 1H), 7.57–7.48 (m, 2H), 7.19 (d, *J* = 8.3 Hz, 3H), 7.04 (d, *J* = 7.9 Hz, 1H), 6.97 (t, *J* = 7.6 Hz, 1H), 6.70 (d, *J* = 16.3 Hz, 1H), 3.86 (s, 3H). ^13^C NMR (151 MHz, Chloroform-*d*) δ 198.3, 168.2, 162.2, 151.7, 142.6, 141.0, 136.7, 134.0, 130.7, 127.7, 123.5, 121.6, 119.6, 117.9, 111.5, 56.1 (Figures S13 and S14).

((1E,4E)-3-oxopenta-1,4-diene-1,5-diyl)bis(2-methoxy-4,1-phenylene) dinicotinate (**G7**).

Calcd. ESI-MS *m*/*z*: 535.5394 (M-H)^−^; measured: 535.5400. M.P.:108–110 °C. ^1^H NMR (600 MHz, Chloroform-*d*) δ 9.42 (s, 1H), 8.88 (d, *J* = 4.1 Hz, 1H), 8.52 (d, *J* = 7.9 Hz, 1H), 7.56–7.53 (m, 1H), 7.51 (d, *J* = 16.3 Hz, 1H), 7.20 (d, *J* = 13.2 Hz, 3H), 6.70 (d, *J* = 16.3 Hz, 1H), 3.87 (s, 3H). ^13^C NMR (151 MHz, Chloroform-*d*) δ 198.3, 163.0, 153.2, 151.6, 150.9, 142.6, 141.4, 138.7, 134.0, 127.7, 125.8, 124.0, 123.4, 121.7, 111.6, 56.1 (Figures S15 and S16).

### 3.2. Molecular Docking Analysis

**Figure 3:**
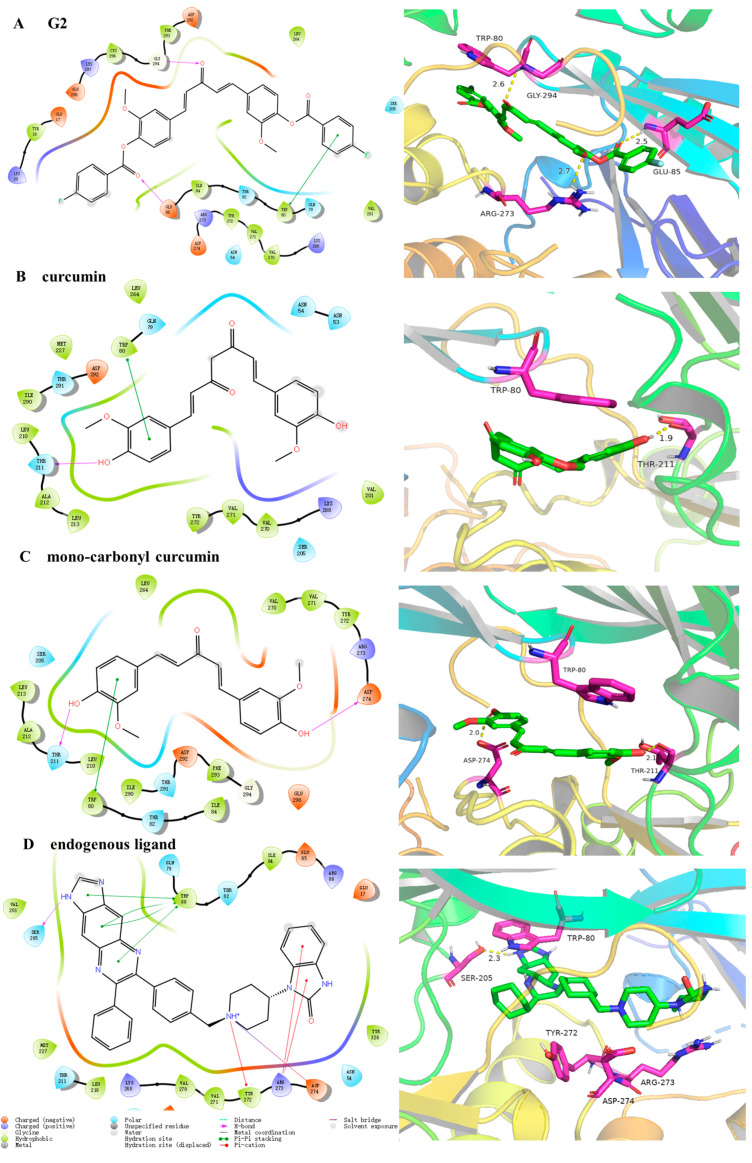
The interaction pattern between derivative **G2** (**A**), curcumin (**B**), mono-carbonyl curcumin (**C**), endogenous ligand (**D**), and AKT protein shows the electrostatic surface in the 2D and 3D representations from molecular docking. **G2** (green) and key residues (red) are represented as sticks and colored by atom type.

To explore the specific binding mode of curcumin and its derivatives with AKT protein, molecular docking was conducted in this study by the Glide module in Schrodinger 2017. The crystal structure of AKT (PDB ID: 3O96, resolution: 2.70 Å, R_free_: 0.308) was downloaded from the Protein Data Bank (www.rcsb.org, accessed on 18 February 2023). The derivatives **G1**–**G7**, curcumin, mono-carbonyl curcumin, and endogenous ligand were docked by standard precision (SP), extra precision (XP) glide, and flexible dock according to the protocols. As shown in Table 1, the docking score of **G1**–**G7** to AKT protein was stronger than endogenous ligand, mono-carbonyl curcumin, and curcumin, playing a pivotal role in protein inhibition and stability no matter the SP or XP method. Importantly, the docking score of **G2** was the highest among all of the derivatives, which was consistent with the result of the MTT assay. As shown in Figure 3, compound **G2** was well extended into the active pocket, which was consistent with the result of the MTT assay and docking score. **G2** was bound to key residues GLU85 (2.5 Å), ARG273 (2.7 Å), and GLY294 (2.6 Å) of AKT through intermolecular hydrogen bonds and formed π–π stacking with key residue TRP80. **G2** formed the same intermolecular forces with key residues TRP80 above the plane of the molecule and TYR272, ARG273, and ASP274 below the plane, when compared to the endogenous ligand. Although **G2** cannot form hydrogen bonds with residue SER205 the same as the endogenous ligand, compound **G2** formed a stronger interaction with key residues GLY294 and GLU85 than the endogenous ligand in the active pocket and the number of key residues of **G2** was more than the parent compounds curcumin and mono-carbonyl curcumin. In addition, the lack of hydrogen bonds with key residues TYR272, ARG273, and ASP274 explained that the overall potency of parents was lower than compound **G2**. These molecular docking results may contribute to the differences in inhibitory activity.

### 3.3. Inhibition Effect on Cell Proliferation

**Figure 4:**
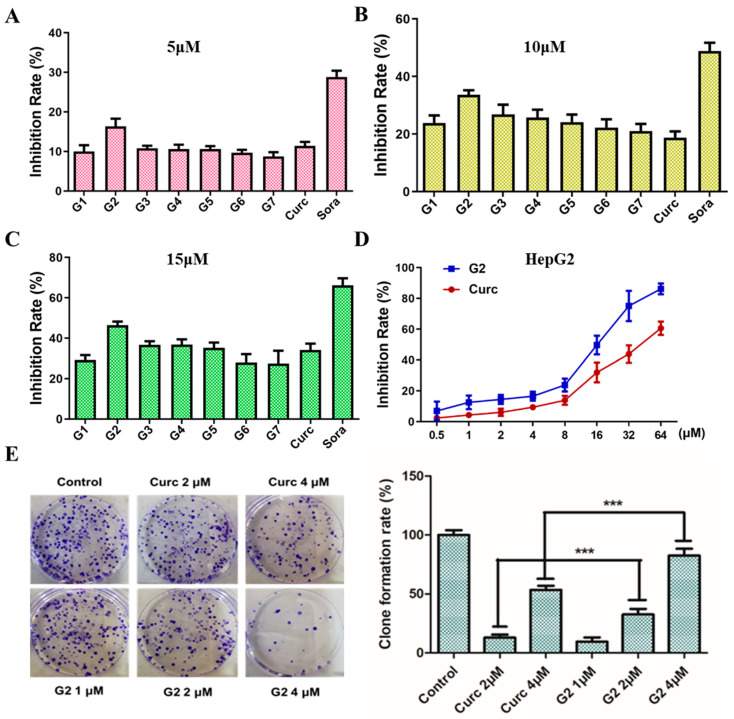
Anti-proliferation assay to HepG2 cells in vitro. (**A**–**C**) Curcumin derivatives **G1**–**G7** at the concentrations of 5, 10, 15 μM for 48 h compared with curcumin and sorafenib. (**D**) **G2** and curcumin at different concentrations (0.5, 1, 2, 4, 8, 16, 32, 64 μM) for 48 h. (**E**) Clone formation assay to HepG2 cells treatment with **G2** (0, 1, 2, 4 μM) for 2 weeks. Data are represented by the mean ± SD of three independent experiments. *** *p* < 0.001, compared with the control group.

The cell proliferation activity of curcumin derivatives **G1**–**G7** towards HepG2 cells was evaluated via MTT assay and clone formation assay. As shown in Figure 4A–C, the cell monolayer was incubated with curcumin derivatives **G1**–**G7** at three concentrations of 5, 10, and 15 μM for 48 h. Curcumin and sorafenib (Sora) were used as positive controls. It was obvious that most of these compounds exhibit more potent toxicity in HepG2 cells when compared with the blank control (only cells sap added group) and the curcumin group. Remarkably, derivative **G2** was the most active at different doses and may be chosen for further studies. The structure–activity relationship (SAR) was discussed briefly: (1) substituents on the C-4 position of the benzyl rings exhibited more effective inhibitory activity than the substituents on the C-2 position comparing **G2** to **G6**; (2) the contribution of the inductive effect of the halogens (such as F, Cl, Br) to anticancer activity was demonstrated by the differences in the activity of compounds **G2**, **G3**, and **G4** with compounds **G1**, **G5**, and **G6**; (3) introducing a carbon ring caused a more significant inhibition than a heterocyclic ring, comparing **G1** with **G7**. The preliminary results could lay the foundation for further structure optimization, and more curcumin derivatives should be synthesized to enrich the SAR.

As shown in Figure 4D, the cells were treated with **G2** with different concentrations (0.5, 1, 2, 4, 8, 16, 32, 64 μM) for 48 h. The IC_50_ values of **G2** and curcumin were 15.39 and 40.56 μM, respectively, and the anti-proliferation activity of **G2** was about 2.64 times higher than that of curcumin. As shown in Figure 4E, the clone formation assay also displayed that **G2** (0, 1, 2, 4 μM) inhibited the clone formation ability of HepG2 cells in a dose-dependent manner for 48 h. The results of MTT and clone formation assay indicated that **G2** exhibited promising anti-proliferation activity in HepG2 cells.

### 3.4. Inhibition Effect on Cell Migration

**Figure 5:**
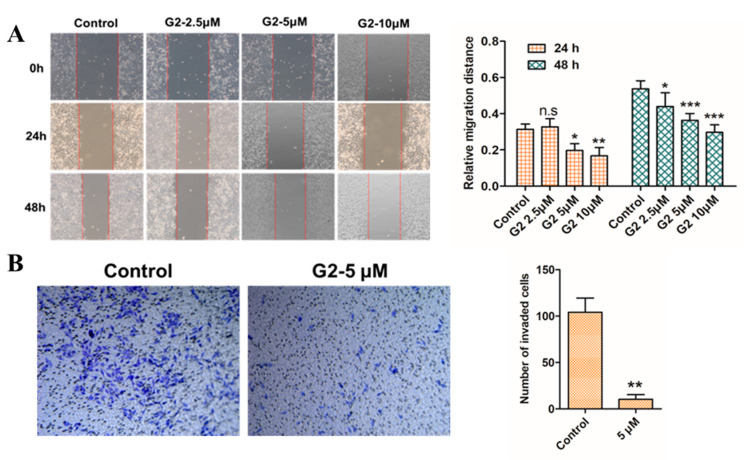
Wound healing assay (**A**) and transwell migration assay (**B**) to HepG2 cells treatment with DMEM medium containing the indicated concentrations (0, 2.5, 5, 10 μM) of **G2** for 24 h and 48 h. Data are represented by the mean ± SD of three independent experiments. *** *p* < 0.001, ** *p* < 0.01, * *p* < 0.05, compared with the 0 μM group.

To verify whether derivative **G2** has an anti-migration effect, a wound healing test and a transwell migration assay were applied to the test. The results of the wound healing assay, shown in Figure 5A, indicated that after treating HepG2 cells with gradient concentrations (0, 2.5, 5, 10 μM) of **G2** for 24 h and 48 h, the relative migration distance of cells was markedly reduced in a dose- and time-dependent manner compared to the control cells. The results of the transwell migration assay in Figure 5B showed that the number of cells traveling through the filter was significantly decreased with **G2** (5 μM) for 48 h. The results of the wound healing test and transwell migration assay together suggested that **G2** exhibited the strong potential to suppress HepG2 cell migration and hence inhibit tumor progression.

### 3.5. Apoptosis-Inducing Effect on HepG2 Cells

**Figure 6:**
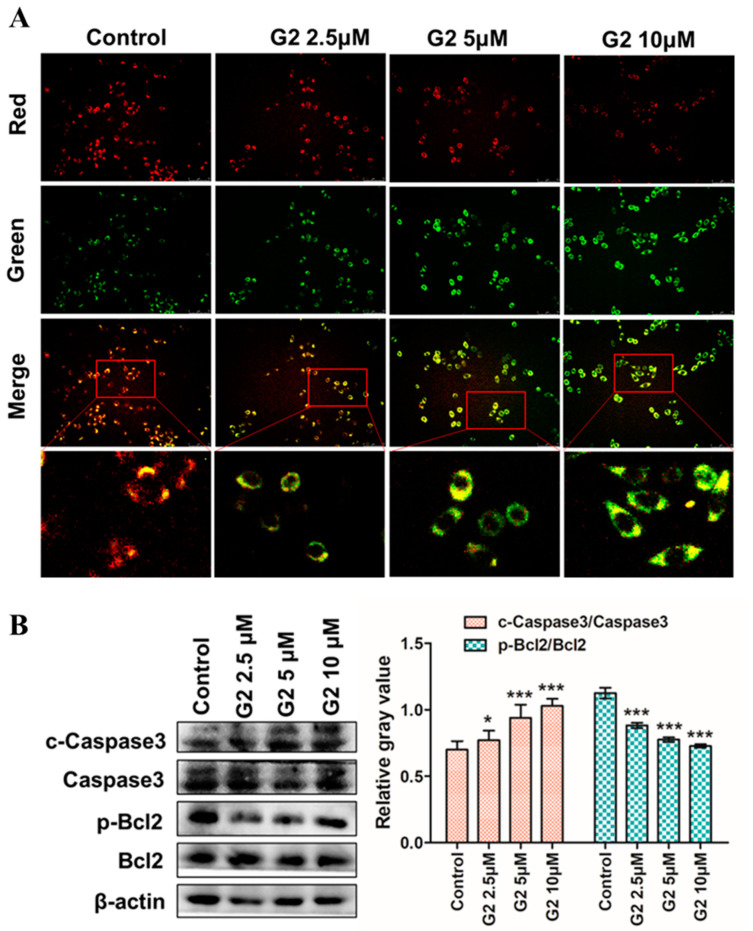
(**A**) Analysis of apoptotic HepG2 cells by JC-1 staining assay. The cell was treated with **G2** (0, 2.5, 5, 10 μM) for 48 h, and then stained with JC-1 for 20 min at 37 °C. The representative images are shown of the corresponding fluorescent channel (40× *g* magnification). (**B**) Apoptosis-related proteins Bcl-2 and caspase 3 protein levels were determined by Western blotting with β-actin as the reference. Data are represented by the mean ± SD of three independent experiments. *** *p* < 0.001, * *p* < 0.05, compared with the 0 μM group.

The depolarization of mitochondrial transmembrane potential (ΔΨ_m_) can be induced via the release of proapoptotic factors when apoptosis happens [44]. Disruption of ΔΨ_m_ can induce the loss of red fluorescence and the gain of green-emitting through JC-1 (5,5′,6,6′-tetrachloro-1,1′,3′,3′-tetraethylbenzimidazolylcarbocyanine iodide) staining [45]. Therefore, we next performed a metachromatic fluorochrome JC-1 staining assay to confirm whether compound **G2** can induce apoptosis. As shown in Figure 6A, HepG2 cells were treated with compound **G2** (0, 2.5, 5, 10 μM) for 48 h and then stained with JC-1 for 20 min at 37 °C. Both the loss of red fluorescence and the gain of green-emitting monomers indicated the disruption of the ΔΨ_m_ in a dose-dependent manner. As shown in Figure 6B, compound **G2** (0, 2.5, 5, 10 μM) caused a significant increase in caspase-3 activation and inhibition of Bcl-2 phosphorylation by Western blotting with β-actin as the reference. These data demonstrated that compound **G2** induced apoptosis in the HepG2 cell in a dose-dependent manner via depolarizing mitochondrial transmembrane potential (ΔΨ_m_).

### 3.6. AKT Inhibition

**Figure 7:**
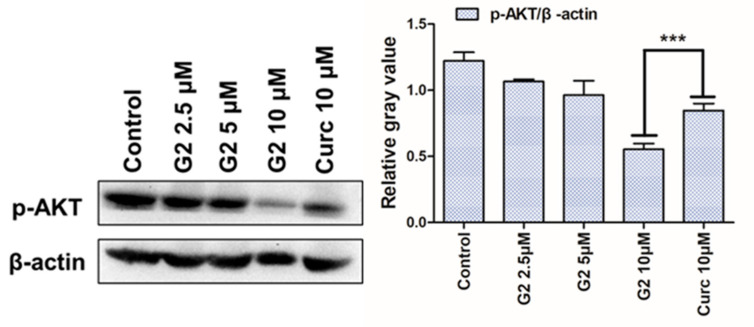
Effect of derivative **G2** (0, 2.5, 5, 10 μM) on Akt protein at the cell level via Western blot. Data are represented by the mean ± SD of three independent experiments. *** *p* < 0.001, compared with the 0 μM group.

Previous studies revealed that curcumin suppressed HCC cancer development through inhibition of the expression of AKT protein [31,46,47]. To further assess whether derivative **G2** had a similar blocking AKT function, the cells were treated with different concentrations (0, 2.5, 5, 10 μM) of **G2** for 48 h, and cell lysates were prepared and analyzed by Western blot. As shown in Figure 7, **G2** markedly suppressed the phosphorylation of AKT in a dose-dependent manner in HepG2 cells compared with curcumin. This result indicated that curcumin derivative **G2** could inhibit the phosphorylation of AKT at the cell level.

### 3.7. Antitumor Activity In Vivo

**Figure 8:**
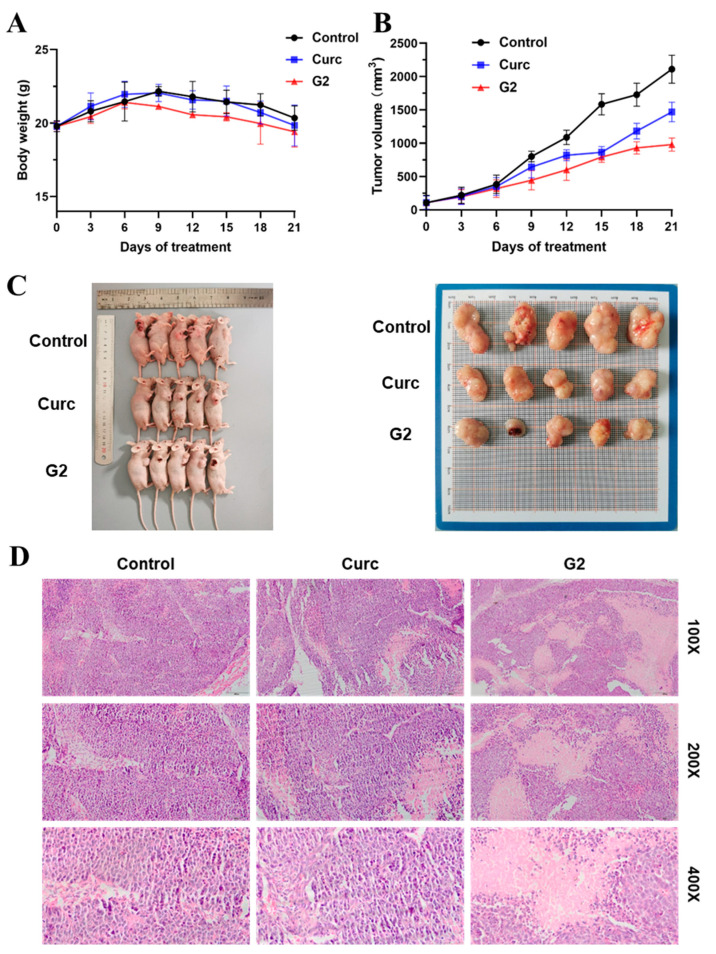
Derivative **G2** inhibited HepG2 xenograft growth in vivo compared with control and curcumin. Body weight (**A**) and tumor volume (**B**) changes in mice were examined every 3 days for 21 days during treatment. (**C**) Visible tumor formation and photographs of representative tumors removed from mice after treatment. (**D**) The xenograft tumor tissues of nude mice were observed and photographed through H&E staining (100×, 200×, and 400×). Data are represented by the mean ± SD of three independent experiments.

Due to the remarkable anti-proliferative, anti-migration, and apoptosis induction activities of **G2** in vitro, its anticancer activity in vivo was assessed utilizing a mouse hepatic carcinoma cancer xenograft model established by the subcutaneous inoculation of HepG2 cells. When the tumor volumes reached approximately 100 mm^3^, the mice were randomized into three groups with five mice in each group. Taking an injection of saline as the solvent vehicle control, curcumin (5 mg/kg) and **G2** (5 mg/kg) of 200 μL were injected into mice intraperitoneally administered every day for 21 days. The body weight (Figure 8A) and tumor volume of nude mice (Figure 8B) were recorded every three days. During treatment, all the animals in different treatment groups were kept at relatively stable body weights, suggesting the low toxicity of **G2**. When the tumors were dissected and weighed after compound treatment, the inhibition rate of the average tumor volume of **G2**-treated mice (53.61%) was significantly better than curcumin (30.41%) (Figure 8C). Then, H&E staining of rat liver cells was conducted to observe the difference in cell morphology in different groups of tumor tissues. As illustrated in Figure 8D, the results showed that curcumin derivative **G2** destroyed the cell structure of transplanted tumor tissues, resulting in the blue-stained nucleus disappearing and the cells and tissues necrotizing. Overall, these findings indicated that curcumin derivative **G2** effectively inhibited cancer growth in vivo and represented a promising drug candidate for hepatocellular carcinoma.

## 4. Conclusions

We assessed the biological activity of novel curcumin derivatives, designated as **G1**–**G7**, in an effort to find a leading molecule with strong anti-HCC activity. Remarkably, **G2** exhibited more significant activity (IC_50_ = 15.39 μM) than curcumin (IC_50_ = 40.56 μM) via MTT assay in HepG2 cells. A series of pharmacological assays containing cell proliferation by clone formation assay, cell migration by wound healing and transwell assay, cell apoptosis by JC-1 staining, and the underlying mechanism were further explored by Western blot and molecular docking. The results showed that **G2** exerted greater potential than curcumin against liver cancer in in vitro studies. Additionally, data from the HepG2 cell xenograft model and H&E staining suggested that the curcumin derivative **G2** had a superior therapeutic impact on tumor development. All in all, **G2** was discovered and identified as a potent anti-hepatoma agent and a new curcumin-like candidate, to be further evaluated in subsequent studies.

## Figures and Tables

**Table 1:** Docking results of SP and XP models of **G1**–**G7** with AKT protein (3O96).

Compounds	Docking Score (Kcal/mol)		Compounds	Docking Score (Kcal/mol)	
	SP	XP		SP	XP
endogenous ligand	−11.044	−10.519	G5	−9.302	−8.721
G2	−9.922	−9.383	G3	−9.288	−8.871
G4	−9.514	−8.56	G6	−9.08	−8.307
G1	−9.457	−8.256	mono-carbonyl curcumin	−7.369	−7.136
G7	−9.344	−8.674	curcumin	−6.599	−7.094

## Data Availability

Data will be made available upon request.

## Supplementary Materials

The following supporting information can be downloaded at: https://www.mdpi.com/article/10.3390/molecules28196796/s1, Figure S1: ^1^H-NMR spectrum of compound **2**; Figure S2: ^13^C-NMR spectrum of compound **2**; Figure S3: ^1^H-NMR spectrum of compound **G1**; Figure S4: ^13^C-NMR spectrum of compound **G1**; Figure S5: ^1^H-NMR spectrum of compound **G2**; Figure S6: ^13^C-NMR spectrum of compound **G2**; Figure S7: ^1^H-NMR spectrum of compound **G3**; Figure S8: ^13^C-NMR spectrum of compound **G3**; Figure S9: ^1^H-NMR spectrum of compound **G4**; Figure S10: ^13^C-NMR spectrum of compound **G4**; Figure S11: ^1^H-NMR spectrum of compound **G5**; Figure S12: ^13^C-NMR spectrum of compound **G5**; Figure S13: ^1^H-NMR spectrum of compound **G6**; Figure S14: ^13^C-NMR spectrum of compound **G6**; Figure S15: ^1^H-NMR spectrum of compound **G7**; Figure S16: ^13^C-NMR spectrum of compound **G7**.
