# Supplementary material for: Discovery of Novel Mono-Carbonyl Curcumin Derivatives as Potential Anti-Hepatoma Agents
Source: Molecules. 2023 Sep 25;28(19):6796. doi: 10.3390/molecules28196796 (PMC10574324; doi:10.3390/molecules28196796)
Supplement: Supplementary file 1 [file molecules-28-06796-s001.zip › molecules-2584201-SI.pdf]

Discovery of Novel Mono-Carbonyl Curcumin Derivatives as Potential Anti-Hepatoma Agents

Weiya Cao [1,2], Pan Yu [1,2,*], Shilong Yang [3], Zheyu Li [2], Qixuan Zhang [2], Zengge Liu [2] and Hongzhuo Li [2]

[1] College of Public Health, Anhui University of Science and Technology, Hefei 230000, China; sml131415@163.com
[2] College of Medicine, Anhui University of Science and Technology, Huainan 232001, China; 17758194831@163.com (Z.L.); 13753738161@163.com (Q.Z.); 15771326234@163.com (Z.L.); 17767778237@163.com (H.L.)
[3] College of Chemical Engineering, Nanjing Forestry University, Nanjing 210037, China; yshl6072@njfu.edu.cn
* Correspondence: 2020035@aust.edu.cn

^{1}H NMR, ^{13}C NMR of target compounds

(1E,4E)-1,5-bis(4-hydroxy-3-methoxyphenyl)penta-1,4-dien-3-one (**2**)

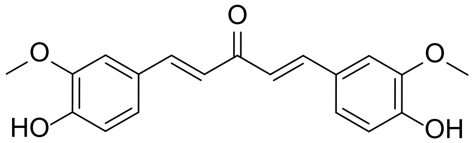

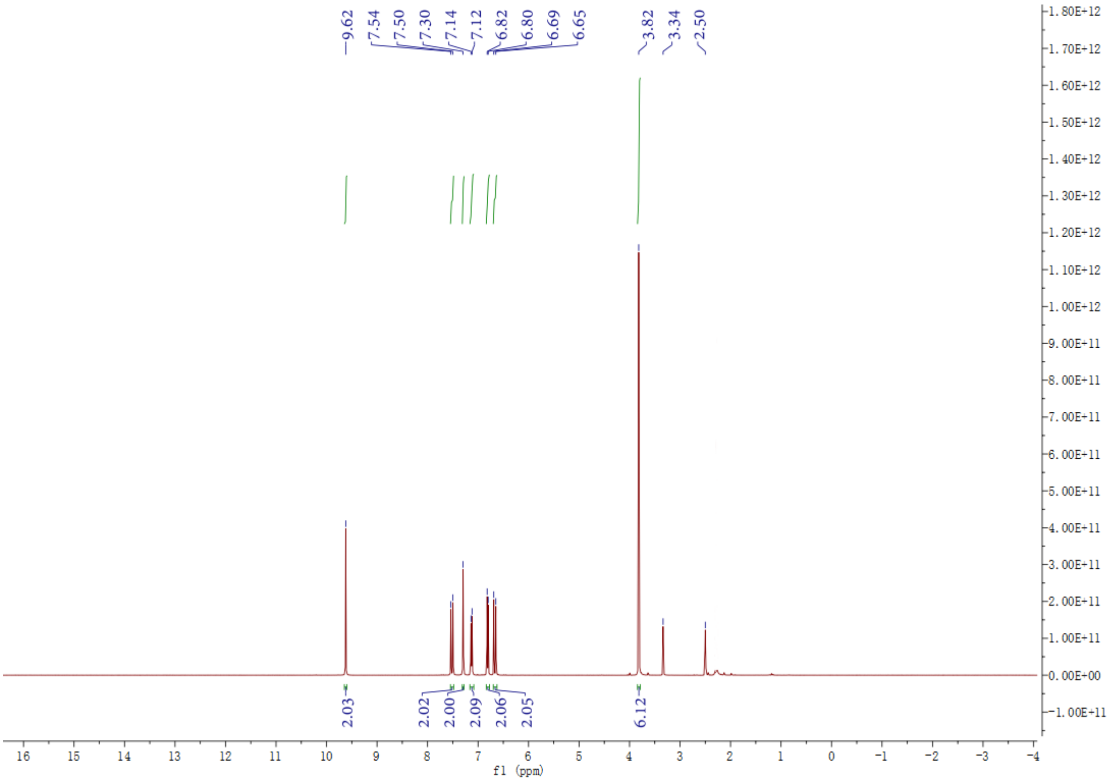

Figure S1. ^{1}H-NMR spectrum of compound **2**

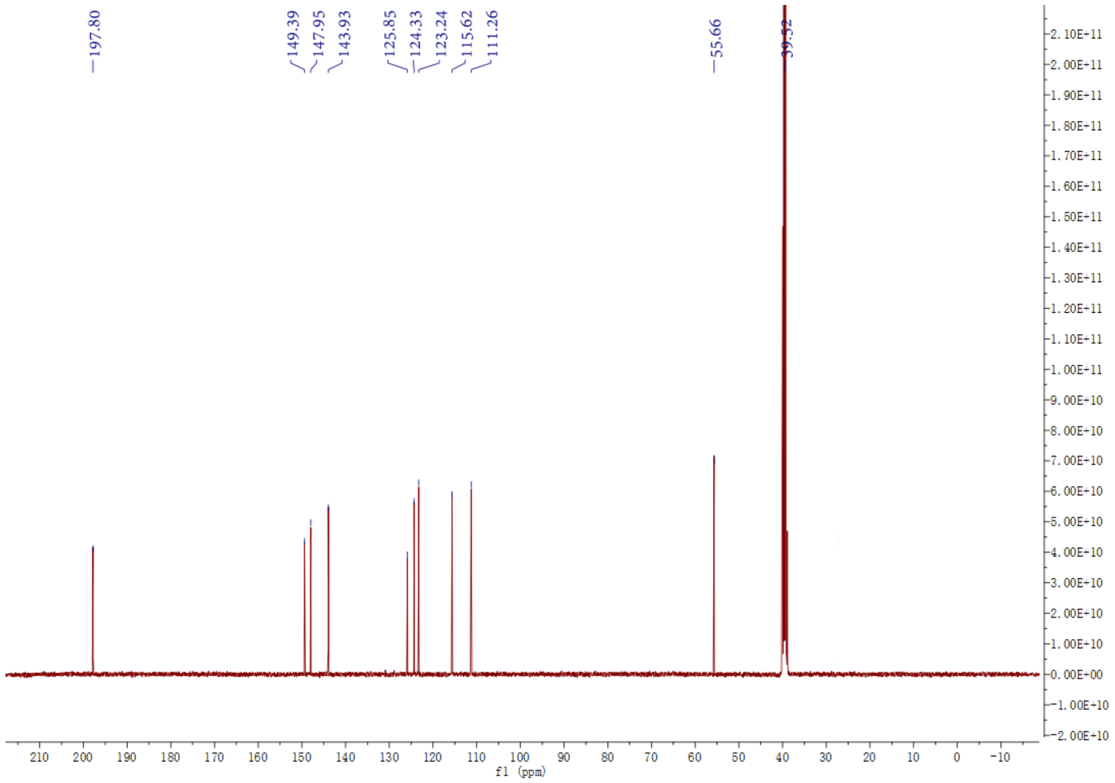

Figure S2. ^{13}C-NMR spectrum of compound **2**

((1E,4E)-3-oxopenta-1,4-diene-1,5-diyl)bis(2-methoxy-4,1-phenylene) dibenzoate (**G1**)

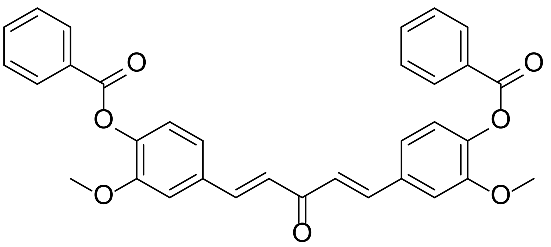

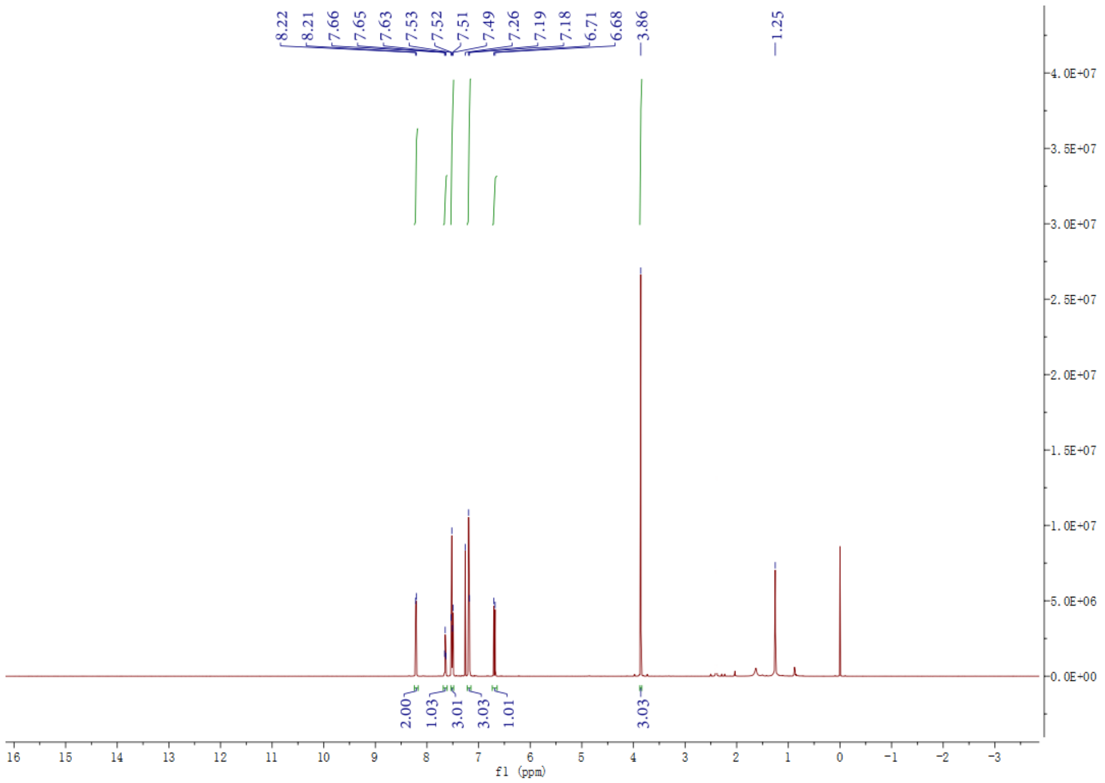

Figure S3. ^{1}H-NMR spectrum of compound **G1**

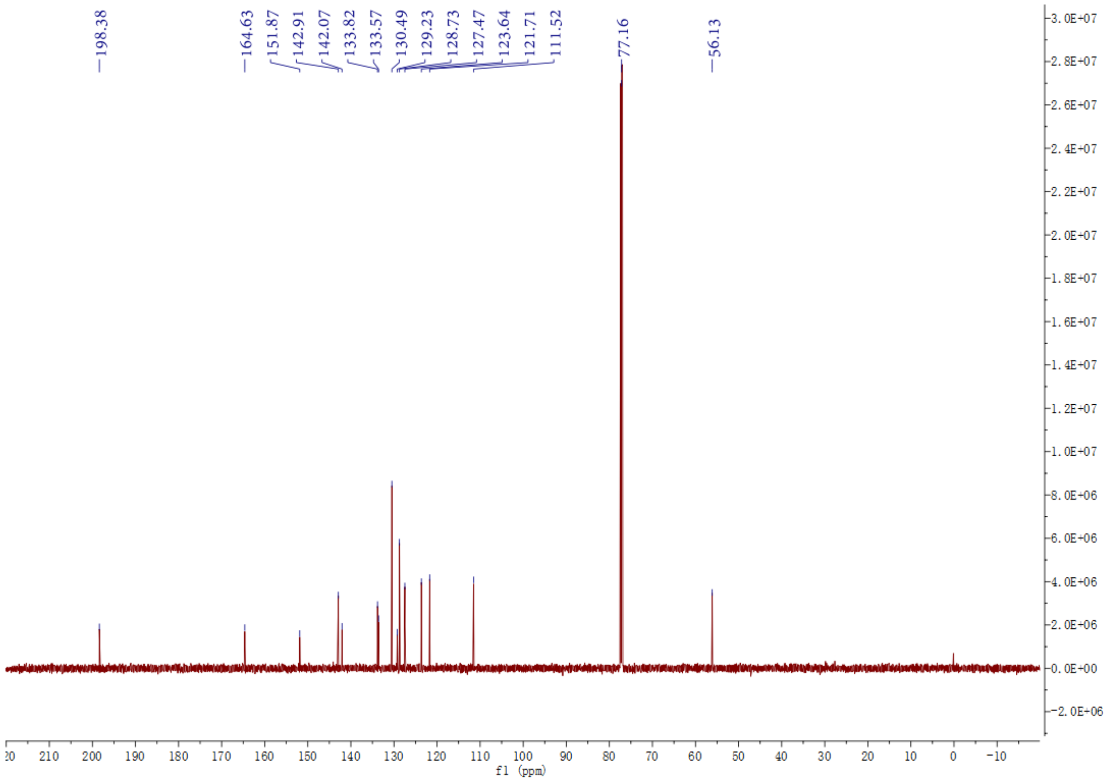

Figure S4. ^{13}C-NMR spectrum of compound **G1**

((1E,4E)-3-oxopenta-1,4-diene-1,5-diyl)bis(2-methoxy-4,1-phenylene) bis(4-fluorobenzoate) (**G2**)

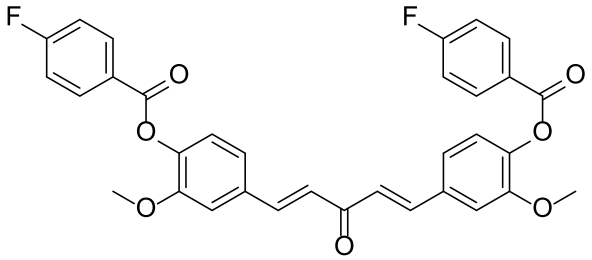

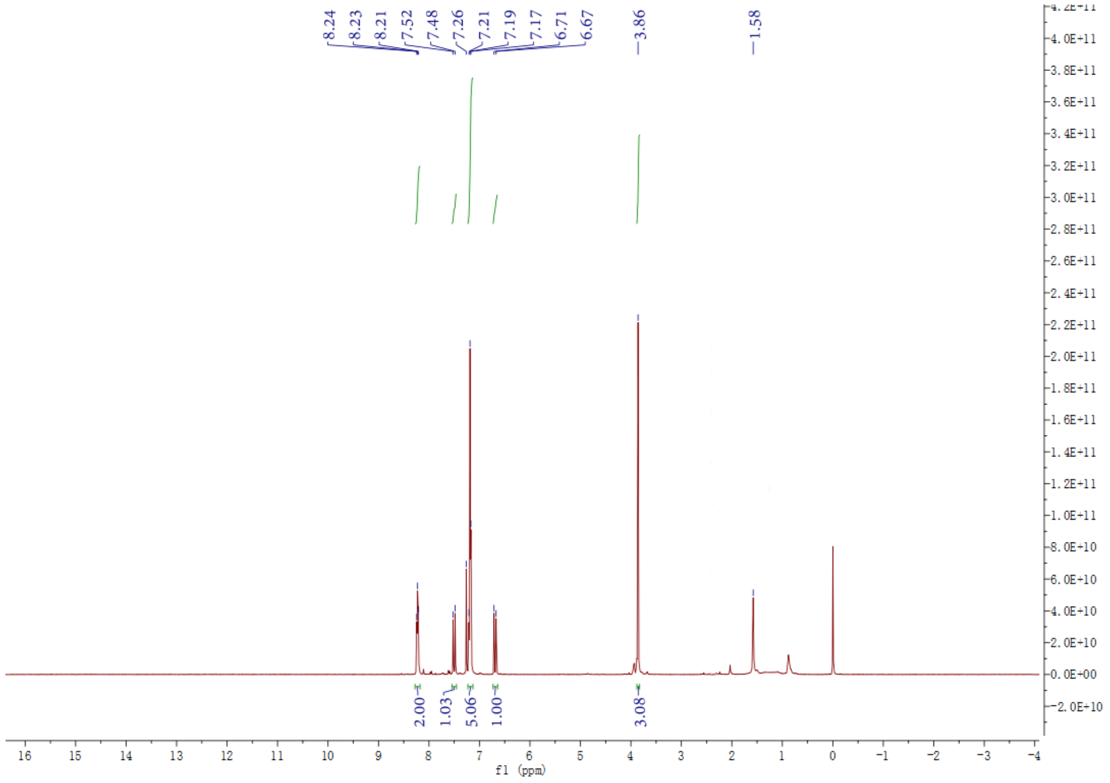

Figure S5. ^{1}H-NMR spectrum of compound **G2**

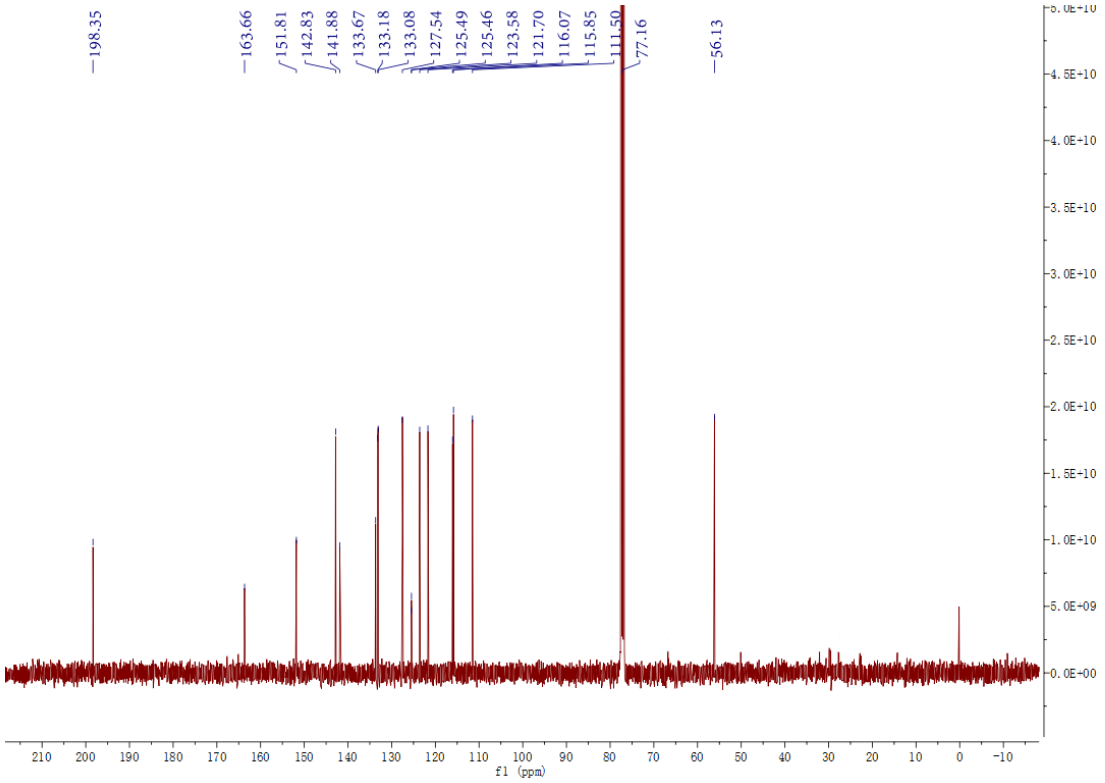

Figure S6. ^{13}C-NMR spectrum of compound **G2**

((1E,4E)-3-oxopenta-1,4-diene-1,5-diyl)bis(2-methoxy-4,1-phenylene) bis(4-chlorobenzoate) (**G3**)

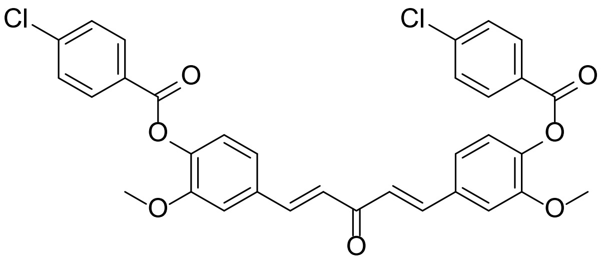

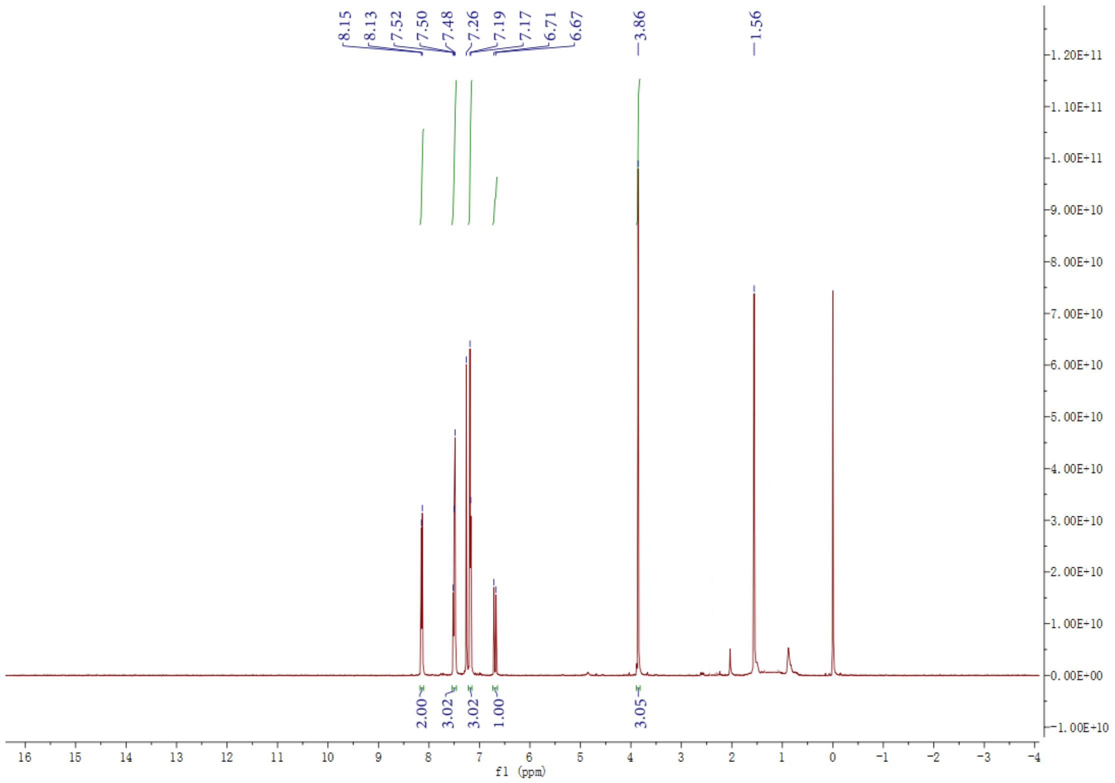

Figure S7. ^{1}H-NMR spectrum of compound **G3**

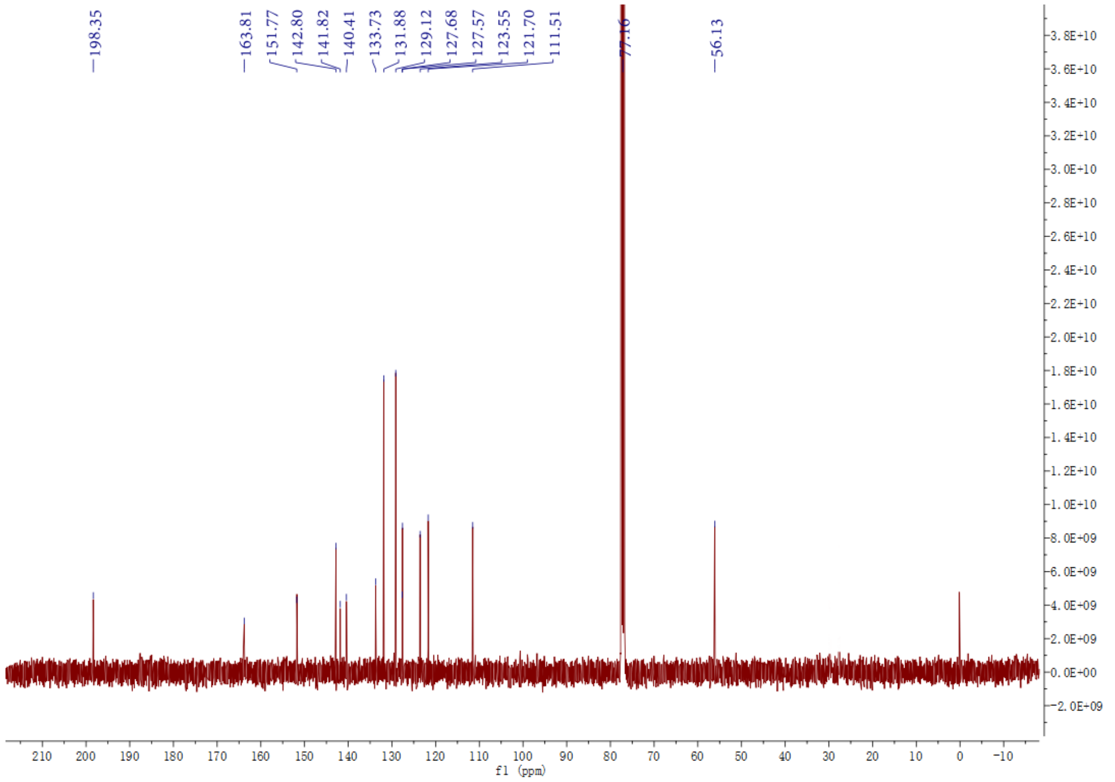

Figure S8. ^{13}C-NMR spectrum of compound **G3**

((1E,4E)-3-oxopenta-1,4-diene-1,5-diyl)bis(2-methoxy-4,1-phenylene) bis(4-bromobenzoate) (**G4**)

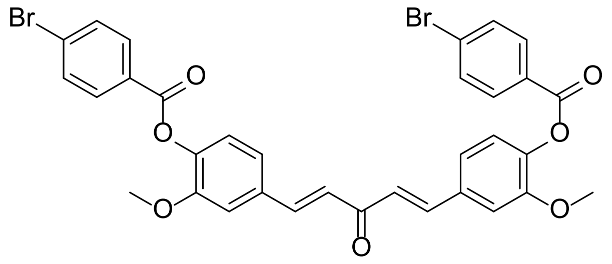

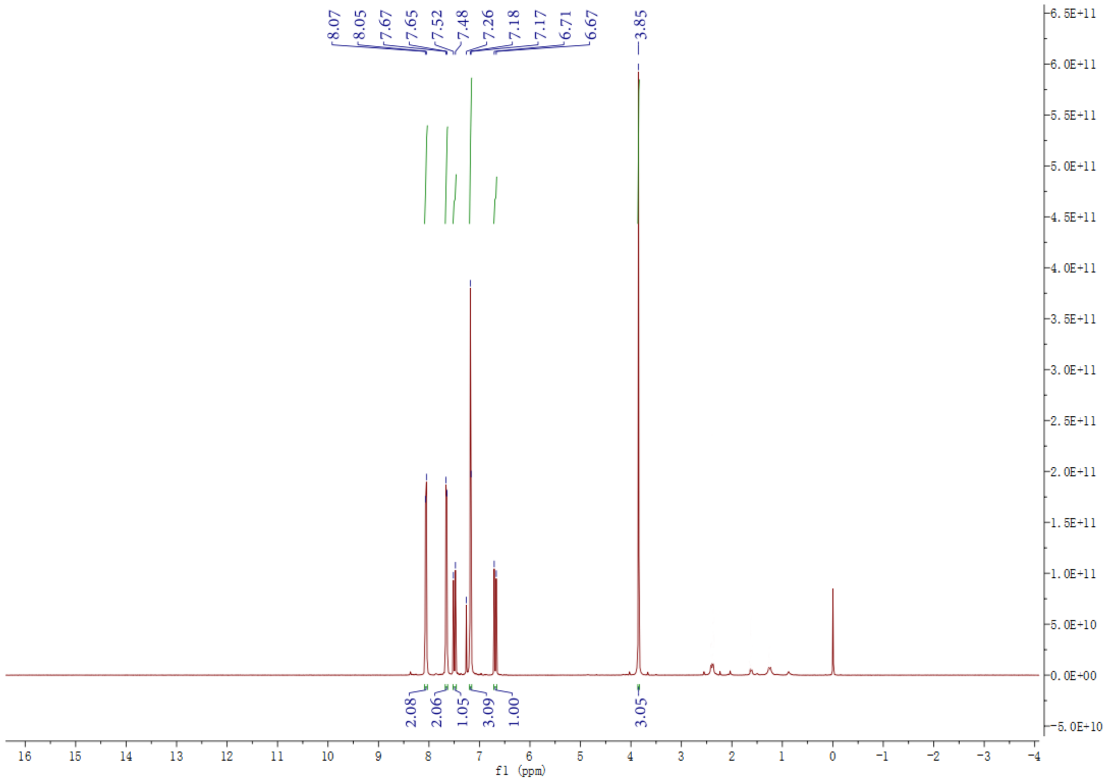

Figure S9. ^{1}H-NMR spectrum of compound **G4**

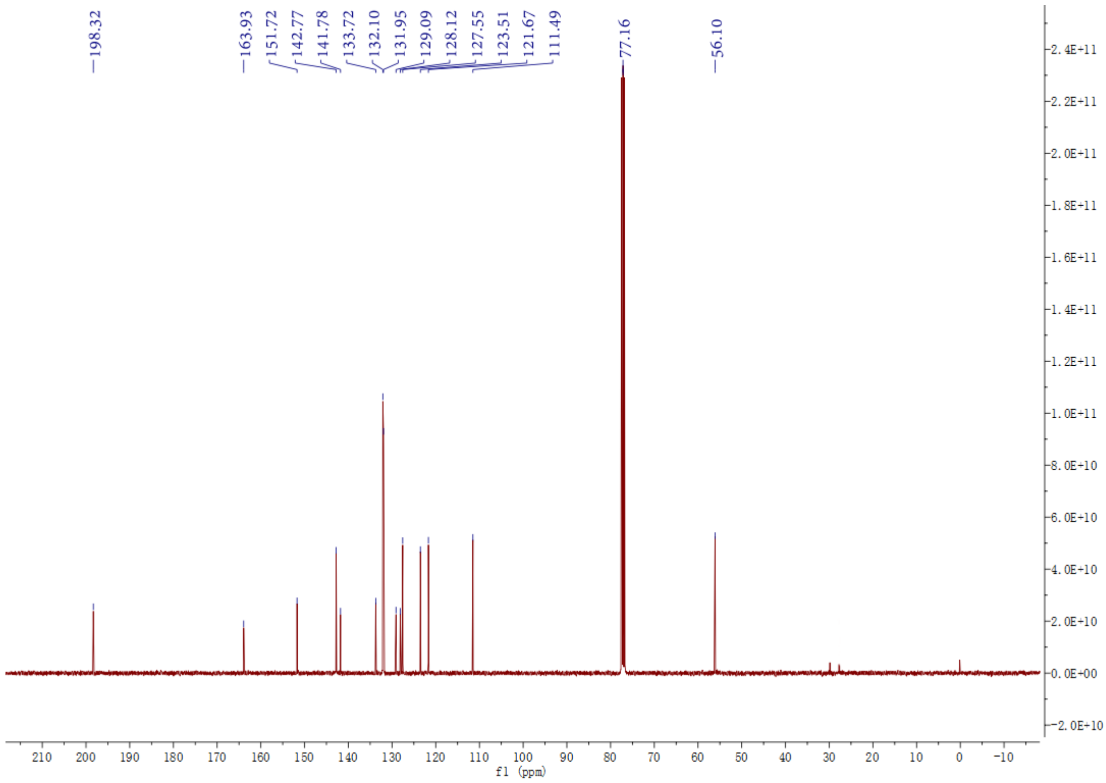

Figure S10. ^{13}C-NMR spectrum of compound **G4**

((1E,4E)-3-oxopenta-1,4-diene-1,5-diyl)bis(2-methoxy-4,1-phenylene) bis(4-(trifluoromethyl)benzoate) (**G5**)

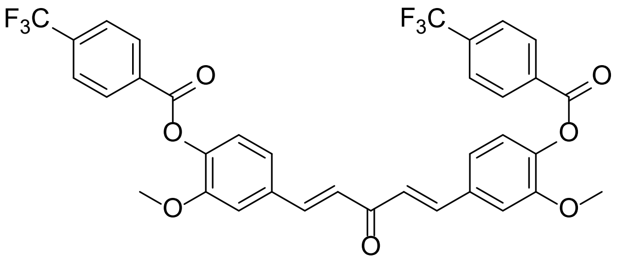

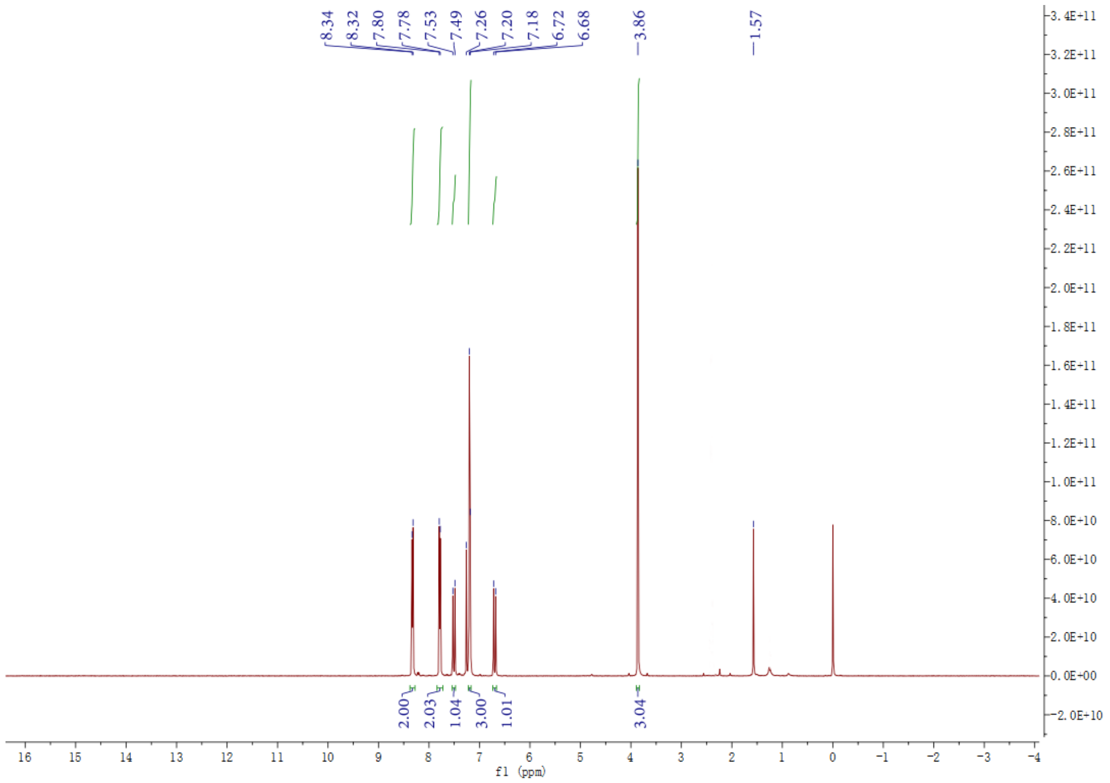

Figure S11. ^{1}H-NMR spectrum of compound **G5**

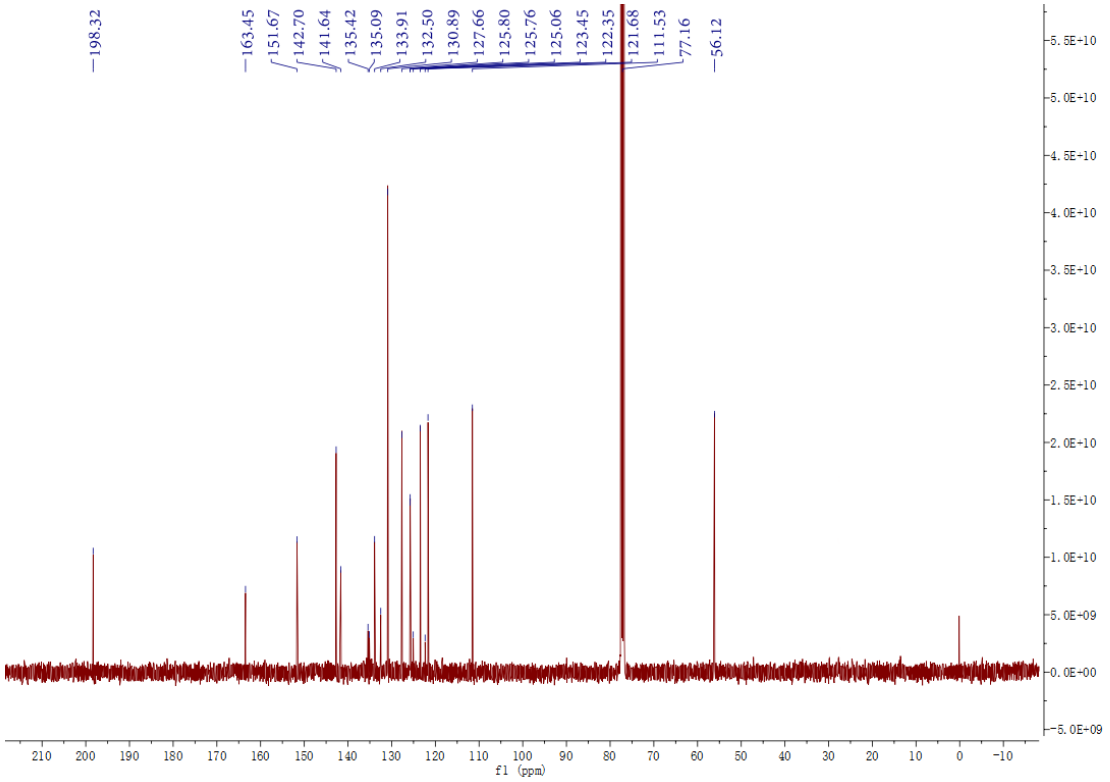

Figure S12. ^{13}C-NMR spectrum of compound **G5**

((1E,4E)-3-oxopenta-1,4-diene-1,5-diyl)bis(2-methoxy-4,1-phenylene) bis(2-hydroxybenzoate) (**G6**)

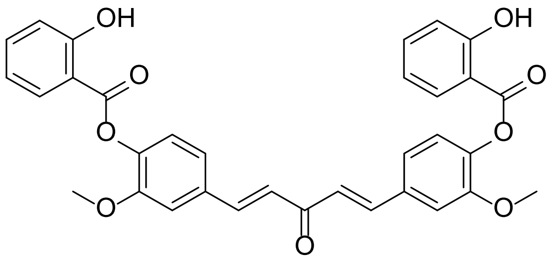

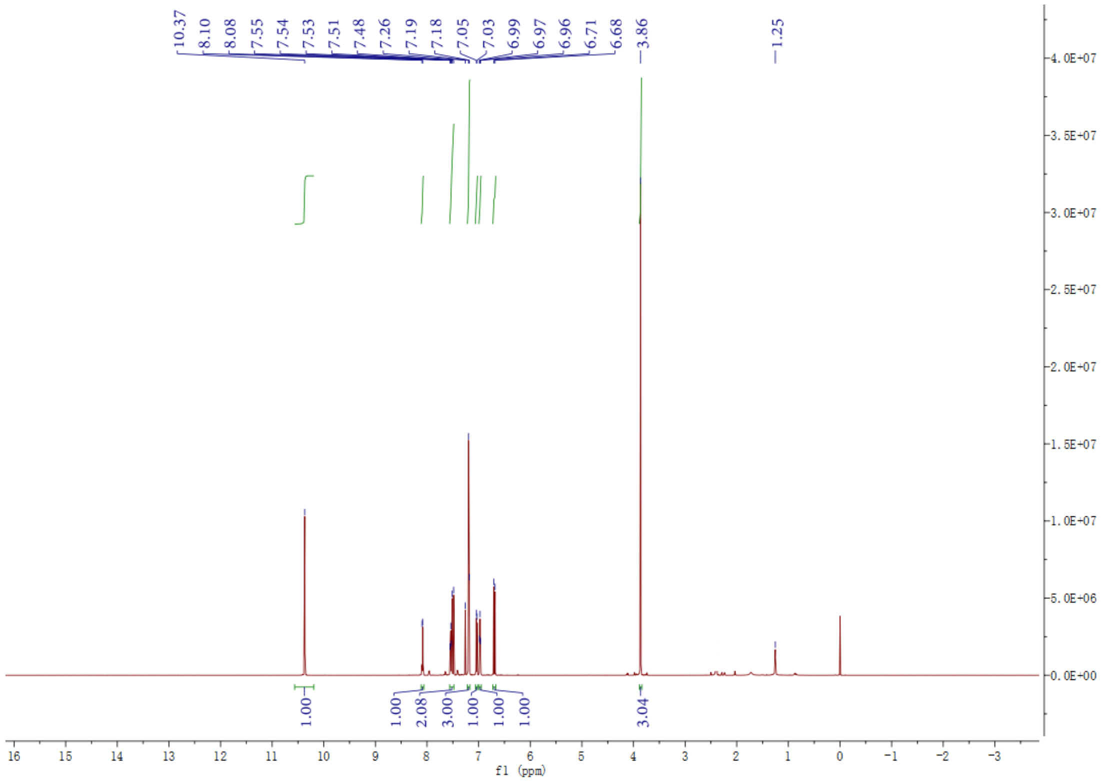

Figure S13. ^{1}H-NMR spectrum of compound **G6**

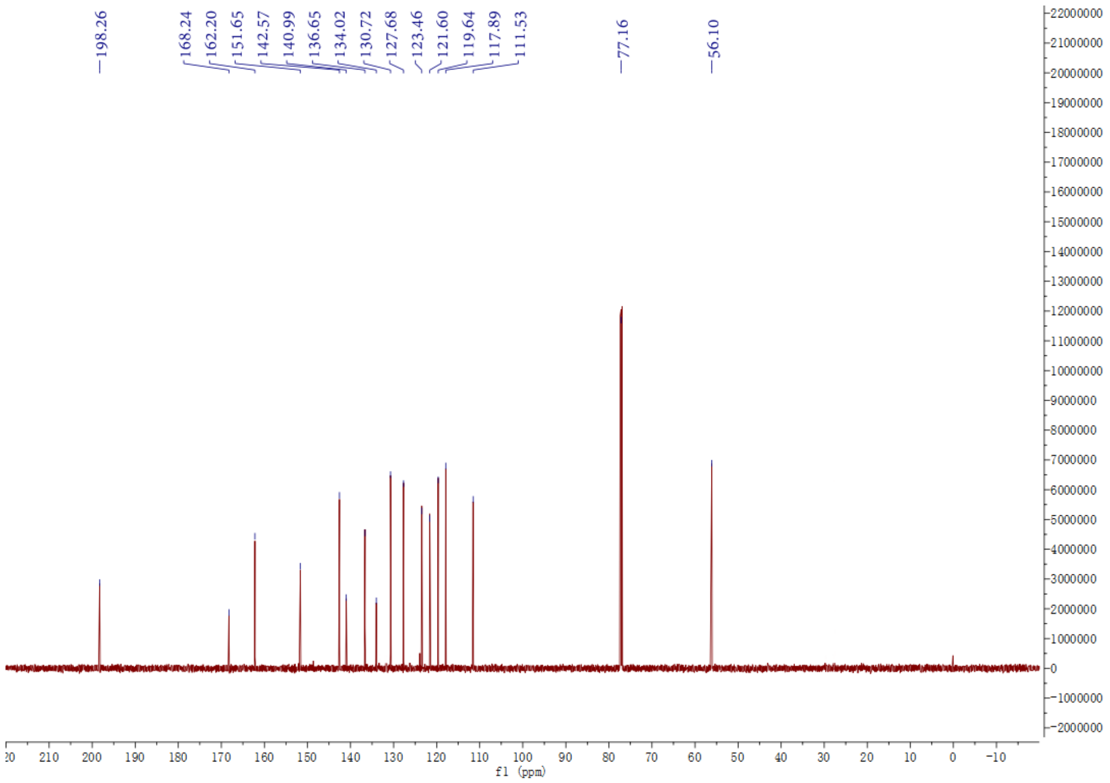

Figure S14. ^{13}C-NMR spectrum of compound **G6**

((1E,4E)-3-oxopenta-1,4-diene-1,5-diyl)bis(2-methoxy-4,1-phenylene) dinicotinate (**G7**)

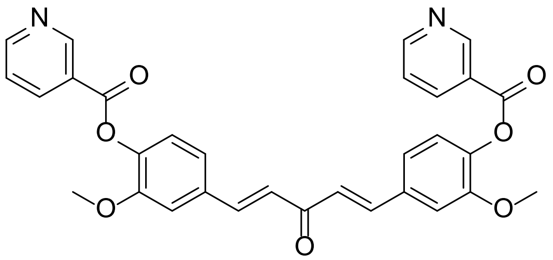

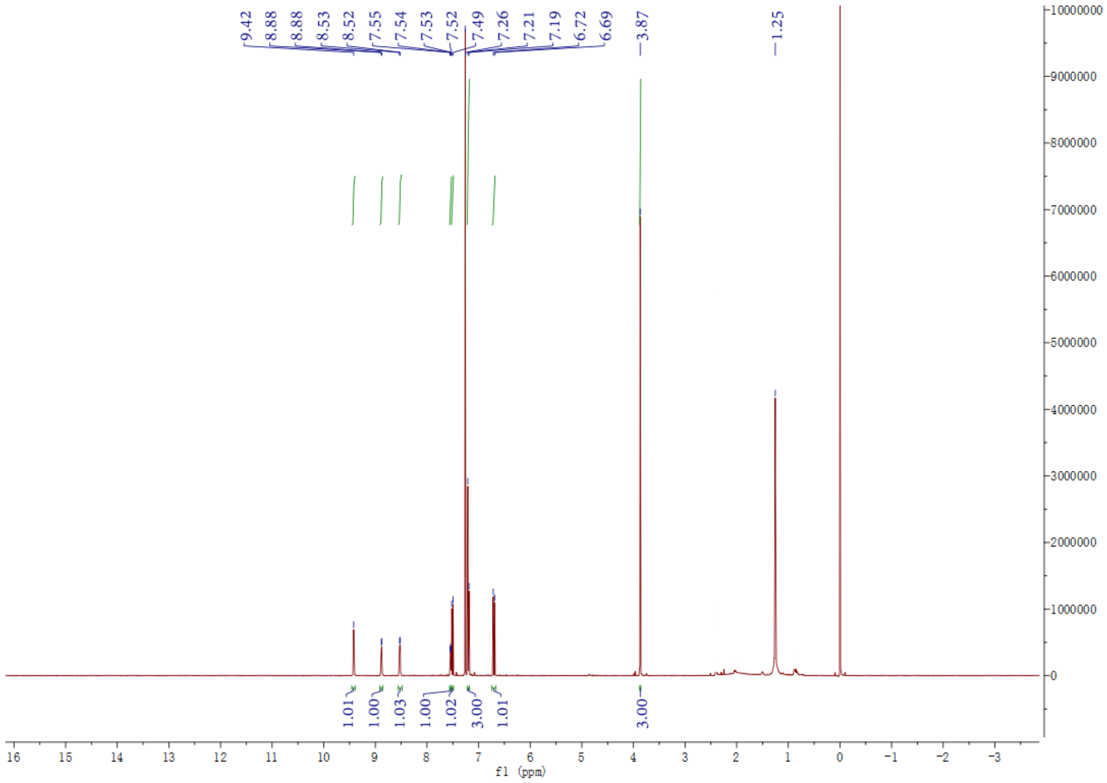

Figure S15. ^{1}H-NMR spectrum of compound **G7**

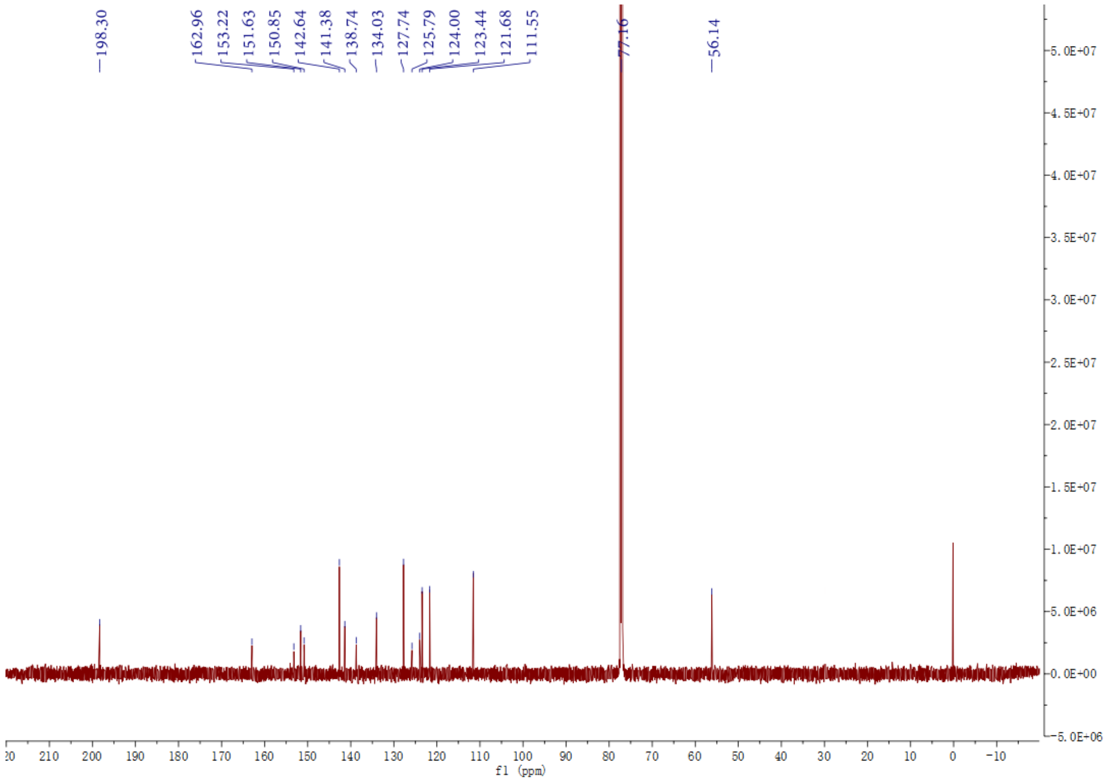

Figure S16. ^{13}C-NMR spectrum of compound **G7**
